# EEG diagnosis of depression based on multi-channel data fusion and clipping augmentation and convolutional neural network

**DOI:** 10.3389/fphys.2022.1029298

**Published:** 2022-10-20

**Authors:** Baiyang Wang, Yuyun Kang, Dongyue Huo, Guifang Feng, Jiawei Zhang, Jiadong Li

**Affiliations:** ^1^ School of Information Science and Engineering, Linyi University, Linyi, China; ^2^ School of Logistics, Linyi University, Linyi, China; ^3^ School of Life Science, Linyi University, Linyi, China; ^4^ International College, Philippine Christian University, Manila, Philippines; ^5^ Linyi Trade Logistics Science and Technology Industry Research Institute, Linyi, China

**Keywords:** multi-channel fusion, depression, CNN, EEG, diagnosis

## Abstract

Depression is an undetectable mental disease. Most of the patients with depressive symptoms do not know that they are suffering from depression. Since the novel Coronavirus pandemic 2019, the number of patients with depression has increased rapidly. There are two kinds of traditional depression diagnosis. One is that professional psychiatrists make diagnosis results for patients, but it is not conducive to large-scale depression detection. Another is to use electroencephalography (EEG) to record neuronal activity. Then, the features of the EEG are extracted using manual or traditional machine learning methods to diagnose the state and type of depression. Although this method achieves good results, it does not fully utilize the multi-channel information of EEG. Aiming at this problem, an EEG diagnosis method for depression based on multi-channel data fusion cropping enhancement and convolutional neural network is proposed. First, the multi-channel EEG data are transformed into 2D images after multi-channel fusion (MCF) and multi-scale clipping (MSC) augmentation. Second, it is trained by a multi-channel convolutional neural network (MCNN). Finally, the trained model is loaded into the detection device to classify the input EEG signals. The experimental results show that the combination of MCF and MSC can make full use of the information contained in the single sensor records, and significantly improve the classification accuracy and clustering effect of depression diagnosis. The method has the advantages of low complexity and good robustness in signal processing and feature extraction, which is beneficial to the wide application of detection systems.

## 1 Introduction

### 1.1 Motivation

With the development of society, the pace of life is getting faster and faster, followed by more and more mental pressure, depression has become a relatively common mental disease. According to the report released by the World Health Organization in 2014, depression has so far affected 350 million people, distributed in different age groups. The proportion of young people suffering from depression is increasing year by year due to the pressure from life, study and employment in many aspects. In recent years, the trend of depression is getting younger (Organization, 2004; Steiger and Pawlowski, 2019; Laacke et al., 2021). Depression is manifested by low mood, lack of confidence and decline in quality of life in the early stage. If not paid attention to and timely treatment, it is likely to develop into major depression, which will lead to suicide attempts of patients and is a very harmful mental disease (Ionescu et al., 2013; Goldberg, 2014). The diagnosis of depression in traditional methods requires professional psychiatrists to make detailed consultation and use the depression self-assessment table to assist diagnosis. This diagnosis method has high accuracy and is the main diagnostic method at present (Neto and Rosa, 2019). However, on the one hand, due to the need for professional psychiatrists, patients need to take the initiative to go to the hospital for diagnosis, which not only requires a high cost of diagnosis, and most of the patients with depression have the psychological rejection of such diagnosis, it is difficult to effectively diagnose patients with depression in the early stage; On the other hand, the recurrence rate of depression after cure is very high, and it is not convenient for patients to conduct effective self-testing to prevent the recurrence of depression (Bilello, 2016; Chan et al., 2018; Roy et al., 2019; Aravena et al., 2020; Ming et al., 2020; Greco et al., 2021).

### 1.2 Related work

In recent years, many researchers have used EEG to automatically recognize human emotions and obtained high recognition accuracy (Kurniawan et al., 2013; Luo et al., 2018; Qing et al., 2019; Torres et al., 2020; Gao et al., 2021; Huang, 2021; Liu et al., 2021; Sharma et al., 2021; Yedukondalu and Sharma, 2022). In Table 1, the methods and accuracy rates of researchers using EEG to diagnose depression are shown.

**TABLE 1:** Comparison with other diagnostic methods for depression.

Reference	Processing	ACC (%)
Cai et al. (2018a) 2018	KNN	79.27
Shon et al. (2018) 2018	GA + KNN	71.76
Acharya et al. (2018) 2018	CNN	96.0
Ay et al. (2019) 2019	CNN + LSTM	99.12
Sharma et al. (2022) 2020	Whale + SVM	97.2559
Akbari et al. (2021) 2021	GA + KNN	99.3
Shon et al. (2018) 2021	CNN + +LSTM	99.1
Proposed work	CNN + +MCF + +MSC	99.63

Lakhan et al. (Sharma et al., 2022) used whale optimization algorithm and support vector machine to identify short-time EEG with an accuracy of 97.2559%. Dongkoo et al. (Shon et al., 2018) used the features selected by the genetic algorithm as the input of the KNN classifier to distinguish the states represented by each EEG data. Akbari et al. (Akbari et al., 2021) extracted geometric features from EEG, used genetic algorithm to reduce the number of feature vector array, and finally used support vector machine to diagnose EEG. Cai et al. (2018a) used a three-electrode EEG system to collect EEG signals from Fp1 (left frontal pole), Fp2 (right frontal pole) and Fpz (mid-frontal pole) electrode positions of subjects. The linear and nonlinear characteristics of EEG were extracted by denoising with kalman derivation formula and discrete wavelet transform.Cai et al. (2020) further fused EEG data of different modes with feature-level fusion technology to establish a more accurate depression recognition model.Chen et al. (2020) analyzed the EEG law of depression by using fuzzy measure entropy. The above methods have been implemented and proved to be effective, but there are still some shortcomings. Firstly, it requires some experience to extract features from EEG. Secondly, the diagnosis process of depression is cumbersome, and it is easy to lose characteristic information, resulting in low classification accuracy and weak generalization ability (Faust et al., 2014; Acharya et al., 2015; Cai et al., 2018b; Sharma et al., 2018; Zhu et al., 2019; Zhu et al., 2020; Sadiq et al., 2021).

Deep learning (DL) algorithm is a new branch of machine learning. It forms more abstract high-level features by combining low-level features, and has the advantages of high precision, automatic feature extraction and selection (Mu and Zeng, 2019). In recent years, deep learning has begun to be used in the diagnosis of depression to classify the EEG of depression (Li et al., 2019; Mumtaz and Qayyum, 2019; Thoduparambil et al., 2020; Dsbah et al., 2021; Seal et al., 2021; Uyulan et al., 2022).Acharya et al. (2018) used the classification method proposed by convolutional neural network for classification, which can conduct automatic and adaptive learning of the input EEG signals to distinguish the EEG of depressed and normal subjects.Ay et al. (2019) used CNN and LSTM architecture to detect depression based on EEG signals. These methods all use EEG signals as the input of CNN directly. Although CNN can produce satisfactory performance in the depression diagnosis, classification accuracy compared with the traditional machine learning methods have made a lot of ascension, however the network training requires a lot of samples to avoid the occurrence of a fitting. In cases where EEG samples for depression are not rich enough, data augmentation techniques are needed to effectively utilize the limited dataset. On the other hand, CNN has unique advantages in image processing. One-dimensional(1D) EEG signals are fused into two-dimensional(2D) images so as to achieve higher recognition accuracy by taking advantage of CNN’s advantages in image processing (Lashgari et al., 2020).

On the basis of existing studies, an EEG diagnosis method for depression based 83 on multi-channel data fusion and clipp augmentation and convolutional neural network is proposed, and gives full play to the role of CNN in depression diagnosis when the data set is small. The specific method is as follows. Firstly, the EEG of healthy and depressed patients was collected, and the original EEG was subjected to multi-scale clipping. Then, the multi-signal and multi-channel fusion method was used to obtain the training data augmented by multi-scale clipping fusion data. Finally, the training data are fed into the multi-channel convolutional neural network training. Compared with the traditional method, the feature extraction process is simplified in the proposed method, and the original EEG signal is converted directly to a two-dimensional image, then the two-dimensional image is used as the input of the neural network for training. The selected depression dataset is described in Section 2. It is introduced in Section 3 that MCF, MSC data augmentation methods and the diagnosis process of depression. In Section 4, experimental results and corresponding analysis are presented to verify the effectiveness of the proposed method. Finally, the conclusion is proposed in Section 5.

## 2 Data description

**FIGURE 1:**
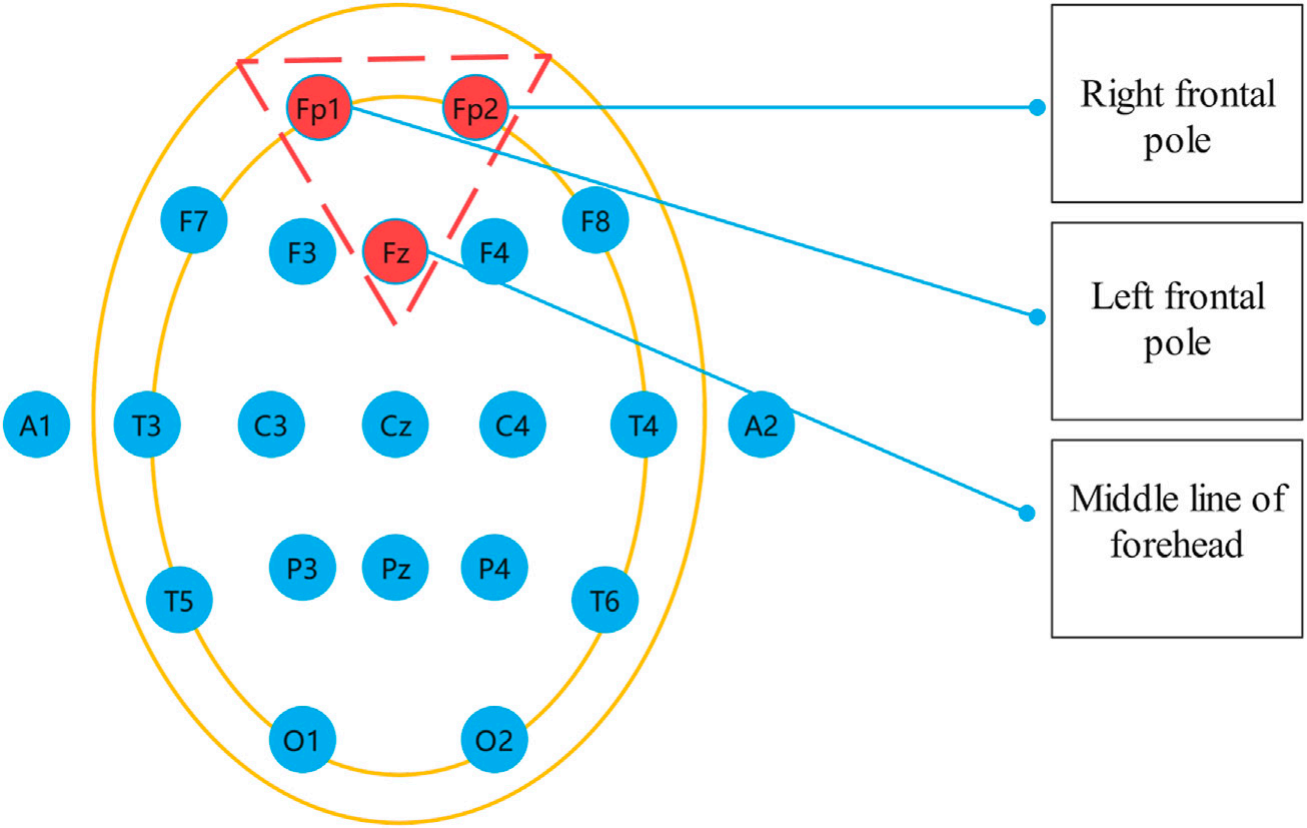
International 10–20 standard.

The international standard for EEG acquisition electrode position in the head is called 10–20 system, which consists of 19 recording electrodes and two reference electrodes, as shown in Figure 1. EEG signals at different positions reflect different functional activities of the brain (Jasper, 1959; Malloy et al., 2006). Therefore, it is necessary to select the appropriate EEG acquisition location when studying depression recognition in EEG. Depression is a psychological disease closely related to emotions. Previous studies have proved that the frontal lobe is the main part of psychological activities and is related to thoughts, emotions and depression, etc. Depression is most closely related to the prefrontal lobe in the frontal lobe (Shi et al., 2020). Therefore, in this study, Fp1, Fp2 and Fpz electrode signals were selected as signal sources to diagnose depression, and MODMA data set from Lanzhou University (Cavanagh et al., 2018) and EEG data set from University of New Mexico (Li et al., 2020) were used to verify the effectiveness of the proposed method in detecting depression EEG.

### 2.1 Dataset 1: MODMA dataset

**FIGURE 2:**
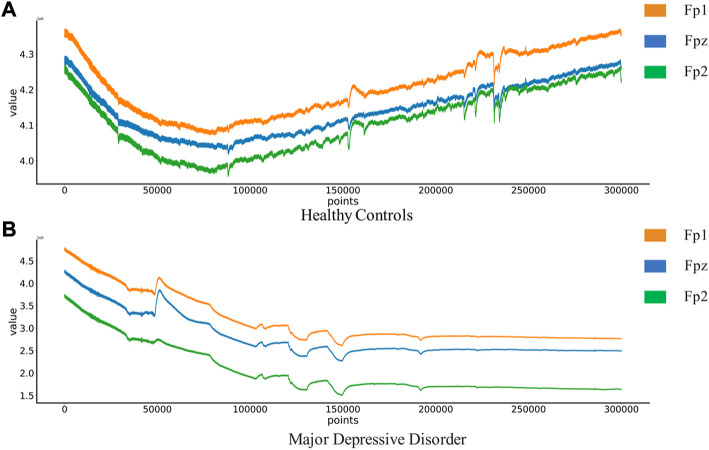
EEG of Healthy Controls and Major Depressive Disorder patients:**(A) **Healthy Controls(HC),**(B)** Major Depressive Disorder(MDD).

The MODMA dataset from Lanzhou University was used as the first dataset. Written informed consent was obtained from all participants prior to the experiment. The local Biomedical Research Ethics Committee of Lanzhou University Second Hospital approved the consent form and study design in accordance with the World Medical Association Code of Ethics (Declaration of Helsinki). The data set included 18 depressed patients and 25 normal controls. The subjects were non-drug users and were aged 18–53. The acquisition device is a three-electrode EEG acquisition sensor, which only collects three electrodes Fp1, Fpz and Fp2. An EEG that records closed eyes and resting states. EEG signals are sampled at 250 hz. After the EEG data was collected, the data quality was evaluated by technicians with EEG processing experience. Below is a EEG of Healthy Controls(HC) and Major Depressive Disorder(MDD) patients in Figure 2.

**FIGURE 3:**
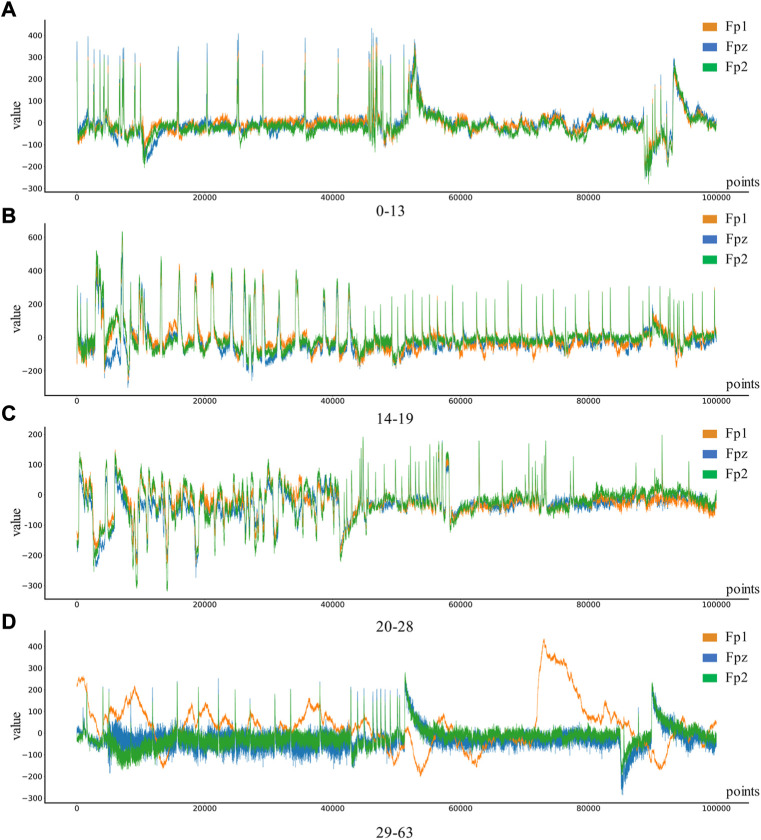
Four different levels of depression: **(A)** 0–13 depression, **(B)** 14–19 depression, **(C)** 20–28 depression, **(D)** 29–63 depression.

Depression Rest dataset of EEG signals are collected from the University of New Mexico. Participants were recruited from introductory Psychology courses based on large-scale survey scores from the Baker Depression Scale (BDI), and all participants in the dataset provided written informed consent forms approved by the University of Arizona. Participants were 18–25 years old, had no history of head trauma or epilepsy, and had no use of psychotropic substances. A score of 0–13 is considered the minimum range for depression, with 14–19 being mild, 20–28 moderate and 29–63 severe. 64 Ag/AgCl electrodes were used to collect EEG signals from the scalp, with bandpass filter 0.5–100 Hz, sampling rate 500 Hz, impedance &lt. 10 k Ω. In this study, only the signals of Fp1, Fp2, and Fpz channels in the dataset will be used to classify and identify the severity of depression. The four types of EEG are shown in Figure 3.

## 3 Data fusion and identification methods

### 3.1 Data Multi-channel Fusion

Convolutional neural network has huge advantages in the field of image recognition. In order to take advantage of the advantages of neural network, it is necessary to fuse the three-channel brainwave signals together and convert them into 2D images, and then use 2D convolutional neural network for direct training and classification. Compared with the direct analysis of the characteristics of three-channel 1D signals this method does not require manual processing and feature extraction of 1D EEG signals.

**FIGURE 4:**
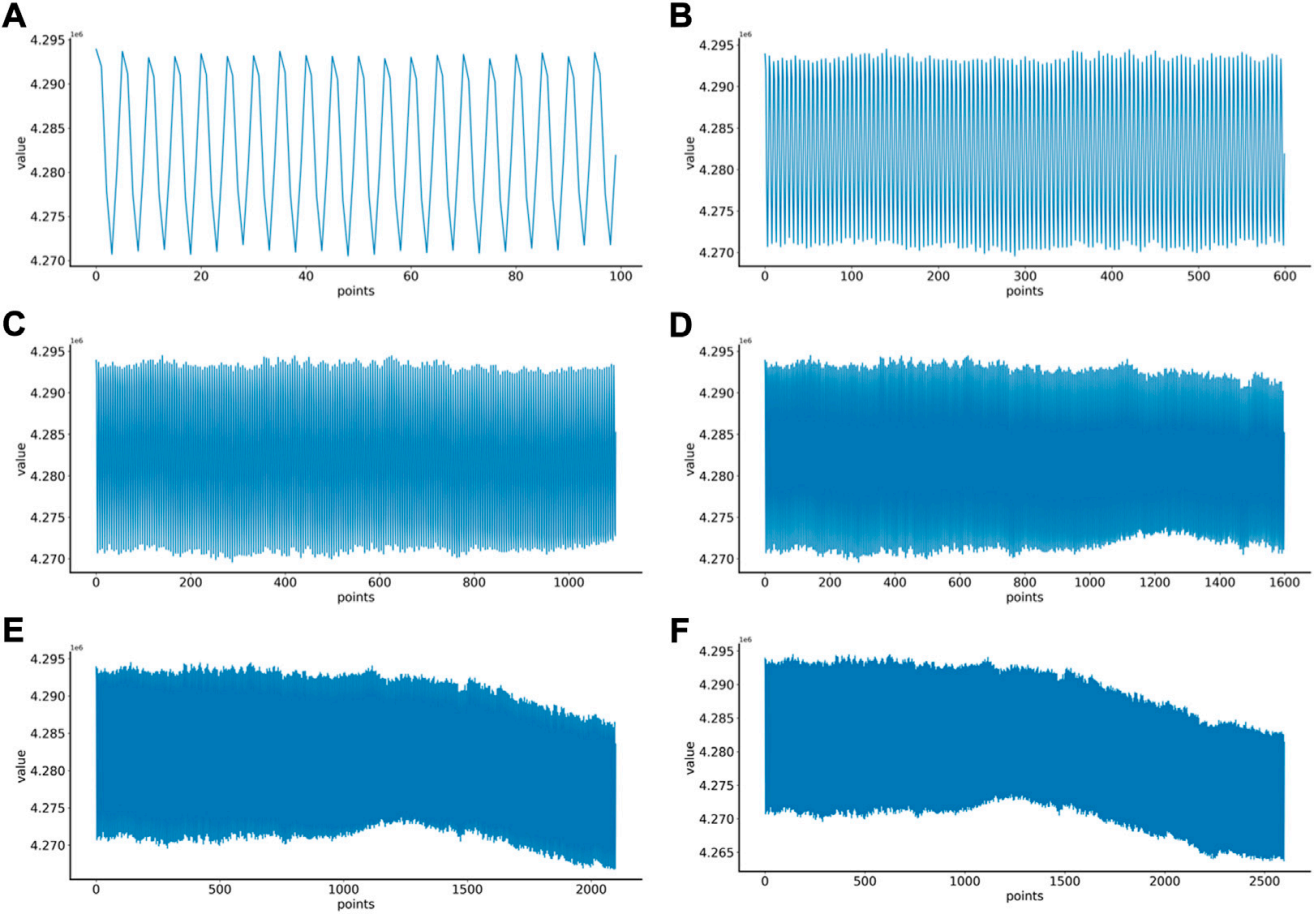
EEG samples of different lengths: **(A)** 100 points, **(B)** 600 points, **(C)** 1100 points, **(D)** 1600 points, **(E)** 2100 points, **(F)** 2600 points.

First, the proper length of a single sample is needed to select, and we make a hypothesis on how to choose an appropriate sample length: When using a convolutional neural network to identify signals, the more data points a single sample contains, the more information it contains. On the contrary, since the number of data points in the original data set is fixed, if each sample contains too many data points, the total number of samples will be too small, which is not conducive to the training of convolutional neural networks. Therefore, in order to select an appropriate sample length, the single-channel data in MODMA dataset were selected and intercepted with the length of 100, 600, 1100, 1600, 2100, and 2600 respectively to draw two-dimensional images, forming a small data set, as shown in Table 2. Samples of different lengths are shown in Figure 4.

**TABLE 2:** Data sets of different sample lengths.

	100	600	1100	1600	2100	2600
HC	2935	2933	2931	2933	2934	2930
MDD	2635	2636	2633	2637	2636	2631

**FIGURE 5:**
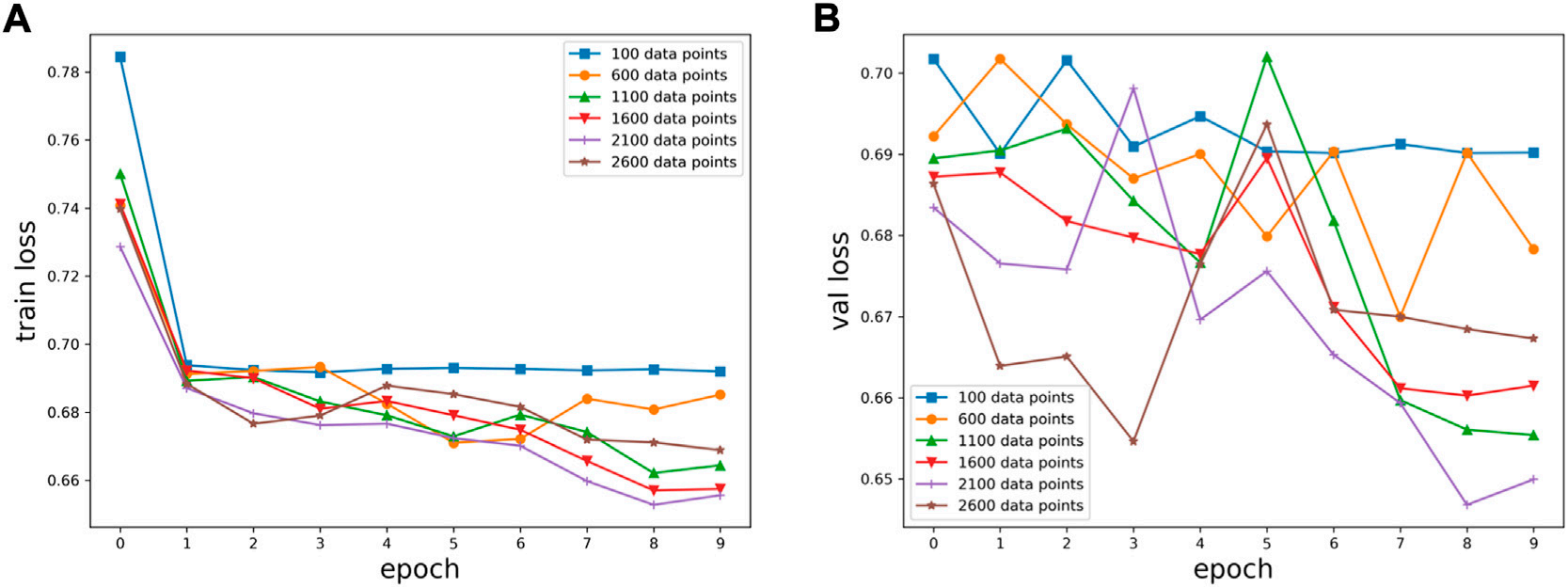
Training results on six datasets of different lengths: **(A)** classification loss function for training set, **(B)** classification loss function for validation set.

**FIGURE 6:**
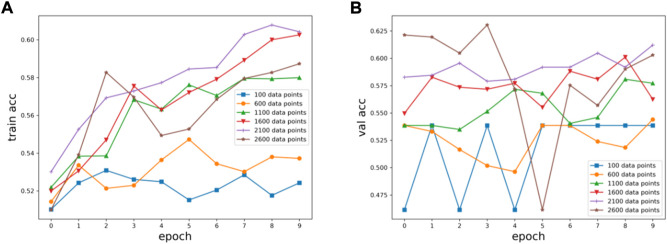
Training results on six datasets of different lengths: **(A)** training set accuracy, **(B)** validation set accuracy.

**FIGURE 7:**
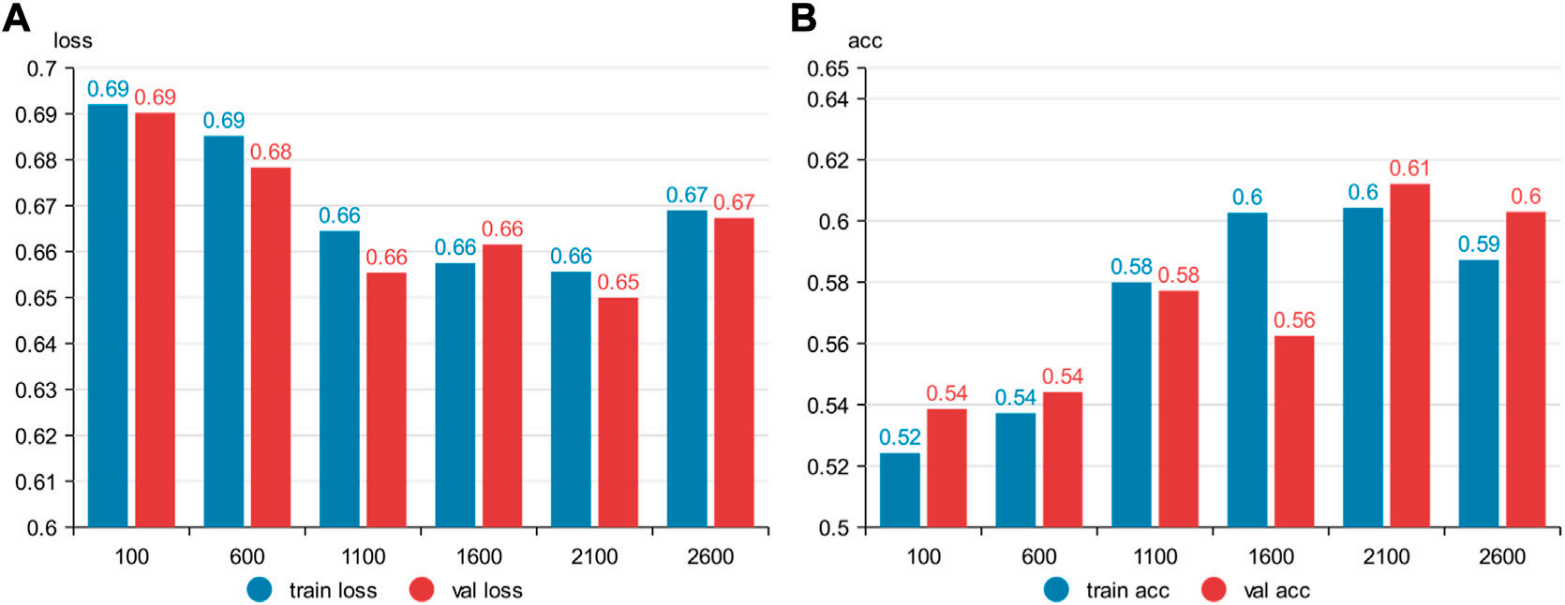
Summary results for six datasets of different lengths: **(A)** accuracy, **(B)** loss function.

Six kinds of single-channel data sets were sent into neural network training, and the EEG accuracy of normal people and depressed patients was distinguished by the results of single-channel EEG training. Finally the data length of a single sample in the data fusion experiment in this paper was determined. The results are shown in Figures 5, 6, 7, respectively. Through the analysis of the results, the general trend is that the more single-sample points contained in the data, the higher the training accuracy. This verifies the conjecture above. But when the data points contained in a single sample reaches 2600, the accuracy begins to decline instead. So considering the size of the data set, as well as the accuracy and smoothness of loss function curve obtained by training, 2100 data points were selected as a sample.

**FIGURE 8:**
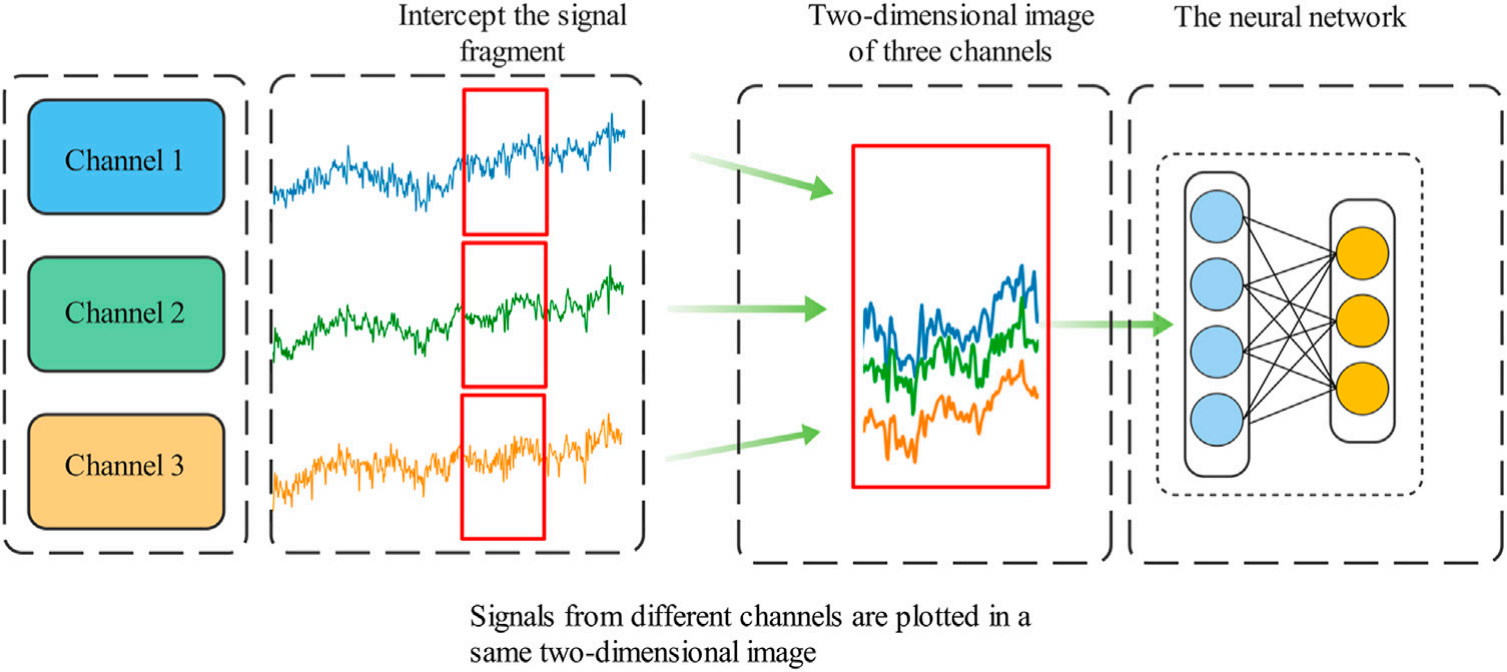
Convolution is inputted after multi-channel data fusion.

A scheme to effectively fuse multi-channel EEG signal information is proposed. The plt function in the Matplotlib package in Python is used to convert the multi-channel data into a 2D image. Before convolution is input, the EEG signals of three channels are fused into a 2D image, which is similar to the visualization of EEG. Compared with single-channel convolution input, it better reflects the association between multi-channel EEG and is easy to expand. This scheme takes 3-channel as an example. However, this method is not limited to 3-channel, and channels can be arbitrarily added or deleted to provide more flexibility, as shown in Figure 8.

### 3.2 Data multi- scale clipping

The collection of EEG data is limited by realistic conditions, and it is very difficult to collect data in a standardized manner, not only because of the limitations of hardware conditions, but also because of the particularity of depression: many depressed patients do not agree to have their EEG data collected. On the other hand, deep learning is based on the method driven by big data. In order to obtain high accuracy and strong generalization ability, it requires multiple types and quantities of data. Inspired by the use of clip, translation, and flip to augment data sets in image processing, EEG can also augment and amplify data sets by translation, which is defined as multi-scale clipping (MSC). Suppose N is the length of the original EEG time series data set, X is the length of a data sample, and C is the augmentation multiple. The number of data samples after data augmentation, M , can be given by the following Formula 1:

$$M=\frac{N}{X}\times C$$ (1)

The sample number of the augmented dataset is increased by C times compared with the original dataset. According to the conclusion in the previous section, the length of a data sample X is 2100, and MSC method is used to expand the original dataset to 2, 4, and 8 times of the original dataset. The specific augmentation steps are as follows:

No data augmentation (AU-N): Take 2100 data points as 1 sample, data 1–2100 is the first sample generated by cutting, the starting point of the next sample is 2101–4200, until the end of the EEG data, and the last time series less than 2100 points are discarded, the definition of each sample can be given by Formula 2, d is the interval of each sample, and S is any one of the M sample sets:

$$S\times X+1\leq d\leq S\times X+X\;S=\{0,1,2,\cdots ,\;\frac{N}{X},\}$$ (2)

times data augmentation (AU-2):Starting from 0 is the original data set, the first sample starts from 1, produces the first sample at 2100, 2101–4200 produces the second sample, and so on, until the end of the sequence data, which is the first part of AU-2; X is 2100 and C is 2, so $\frac{X}{C}$ is 1050 data points. Use 1051 as a starting point, to 3150 is the first sample of part 2, and so on, the second sample is 3151–5150, the third sample is 5151–7250, until the end of the sequence data, data segments less than 2100 will be discarded. The two new data sets are then fused into a data set about twice the size of the original one, known as double data augmentation, or “AU-2”. The definition of each sample can be given by Formula 3, where $\frac{X}{C}$ replaces X in the original formula.

$$S\times \frac{X}{C}+1\leq d\leq S\times \frac{X}{C}+X\;S=\{0,1,2,\cdots ,\;\frac{N}{X}\times C\},C\neq 0$$ (3)

Four times data augmentation(AU-4): The same original data set starts from 1, generates the first sample at 2100, generates the second sample at 2101–4200, and so on until the end of the sequence data, discarding the part of the last segment of the sequence less than 2100. This is part 1 of AU-4; $\frac{X}{C}$ is 525 data points. Part 2 starts from 526, to 2625 is the first sample of Part 2, and so on. The second sample of Part 2 is 2626–4725, and the third sample is 4726–6826, until the end of the sequence data. Part 3 (Samples 1:1051 to 3150, Samples 2: 351 to 5250, samples 3:5251 to 7350); The fourth part is (Sample 1:1576 to 3675, sample 2:3676 to 5775, sample 3:5776 to 7875). These four new data sets are then fused into a data set, that is, approximately four times the size of the original one, which is known as the quad-data augmentation, or AU-4 for short.

Eight times data augmentation(AU-8): The same original data set as part 1 of AU-8; $\frac{X}{C}$ is 262.5.263 data points are selected after rounding. The second part is (Sample 1:264–2363, sample 2:2364–4463, sample 3:4644–6563). The third part is (Sample 1:527–2626, Sample 2: 26.27–4726, sample 3: 47.27–6826). The fourth part is (Sample 1:790–2889, sample 2:2890–4989, sample 3:4990–7090). The remaining four parts of AU-8 are generated according to the above steps, and then the eight new data sets are fused into a data set about 8 times the original data set, which is called 8-fold data augmentation, referred to as “AU-8”.

### 3.3 Convolutional neural network

As an important member of neural network, CNN has powerful capability of representation learning and automatic feature extraction. So far, there are many variants. Mainstream CNN models include AlexNet, GoogLeNet and VGG network, etc., but the basic structure of CNN includes input layer, convolution layer, pooling layer, full connection layer and output layer.

The convolution layer uses the convolution kernel to extract features, and contains multiple convolution kernels. Each neuron is only connected to the local area of the previous layer. This area is called the “receptive field”, and the size of the receptive field depends on the convolution kernel. The Formula 4 is defined as follows, where p×q is the size of the convolution kernel, w is the weight of the convolution kernel, v is the image gray value, and the bias b is added after the convolution; f is the activation function.

$$z_{x,y}=f\left(\overset{p*q}{\underset{i}{\sum }}w_{i}v_{i}+b\right)$$ (4)

Pooling layer is a subsampling operation. Its main objective is to reduce the size of the feature graph. It is carried out by Max Poolling method and its Formula 5 is as follows:

$$f=\mathrm{Max}\left(x_{m,n},x_{m+1,n},x_{m,n+1},x_{m+1,n+1}\right)\left(0\leq m\leq M,0\leq n\leq N\right)$$ (5)

After several iterations of the convolutional layer and the pooling layer, the full connection layer connects the neurons of the first several layers, extracts the nonlinear combination of features and then tiles them into vectors as the input of the final classifier. The output layer (SoftMax) is a general form of logistic regression, which can realize multi-classification problems. For input data $\left\{\left(x_{1},y_{1}\right),\left(x_{2},y_{2}\right),\ldots ,\left(x_{n},y_{n}\right)\right\}$ has k categories, and SoftMax estimates the probability that input data X belongs to each of k categories. In Formula 6: $\theta_{1},\theta_{2},\ldots ,\theta_{k}$ are the learning parameters of the model, multiplied by $\frac{1}{\sum_{j=1}^{k}e^{\theta_{j}^{T}x_{i}}}$ to make the probability distribution between [0,1].

$$h_{\theta }\left(x_{i}\right)=\left[\begin{array}{c} p\left(y_{i}=1|x_{i};\theta \right)\\ p\left(y_{i}=2|x_{i};\theta \right)\\ \vdots \\ p\left(y_{i}=k|x_{i};\theta \right) \end{array}\right]=\frac{1}{\sum_{j=1}^{k}e^{\theta_{j}^{T}x_{i}}}\left[\begin{array}{c} e^{\theta_{1}^{T}x_{i}}\\ e^{\theta_{2}^{T}x_{i}}\\ \vdots \\ e^{\theta_{k}^{T}x_{i}} \end{array}\right]$$ (6)

The classical VGG structure in CNN is used to extract and classify EEG features. VGG is a network proposed by Visual Geometry Group of the University of Oxford in 2014, which contains 13 convolutional layers and three fully connected layers. This network reduces required parameters by stacking multiple 3x3 convolutional kernels to replace large-scale convolutional kernels. Stacking two 3x3 convolutional kernels can replace the sensory fields of 5x5 convolutional kernels. Stacking three 3x3 convolution kernels can replace the receptive field of 7x7 convolution kernels (Simonyan and Zisserman, 2014). The structure of VGG is shown in Table 3.

**TABLE 3:** VGG neural network structure.

Layer	Convolution kernel	Size
1	RGB image	224×224
2	Convolutional layer	64×64
3	Convolutional layer	64×64
4	Maxpool	
5	Convolutional layer	128×128
6	Convolutional layer	128×128
7	Maxpool	
8	Convolutional layer	256×256
9	Convolutional layer	256×256
10	Maxpool	
11	Convolutional layer	512×512
12	Convolutional layer	512×512
13	Convolutional layer	512×512
14	Maxpool	
15	Convolutional layer	512×512
16	Convolutional layer	512×512
17	Convolutional layer	512×512
18	Maxpool	
19	Fully Connected Laye	2048
20	Fully Connected Laye	2048
21	Fully Connected Laye	2
22	Soft-max	

EEG, diagnosis of depression.

**FIGURE 9:**
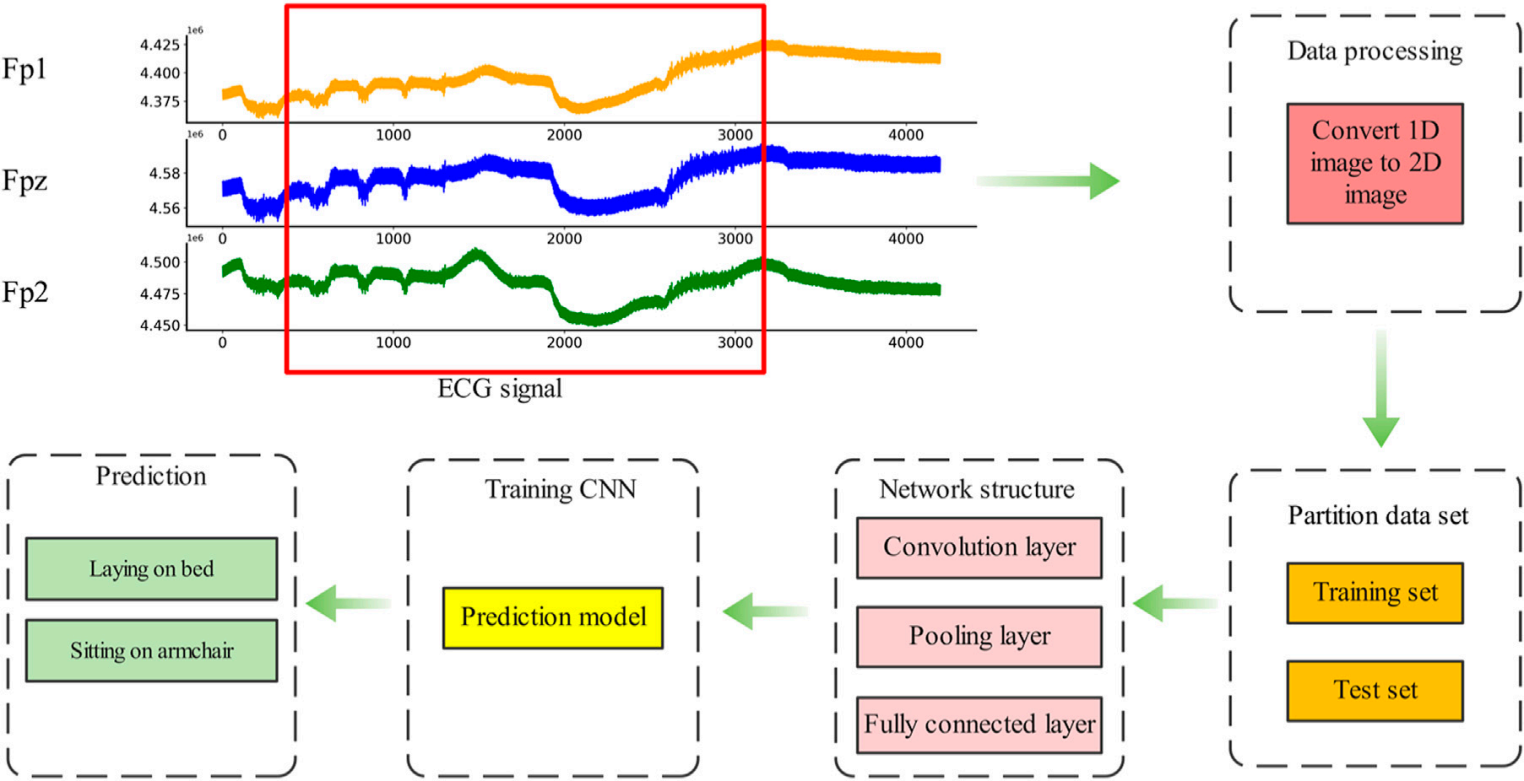
Flowchart of EEG diagnosis of depression.

After multi-channel data fusion and clipp augmentation, convolutional neural network was designed for depression EEG diagnosis. The method was divided into five basic steps, and the flow chart was shown in Figure 9.

Step 1 : EEG signals were collected by wearable EEG acquisition equipment, and selected data sets were used in this study instead of data collection.

Step 2 : Processing the collected EEG signals, Fpz and Fp3 EEG signals are fused to generate the initial data set, and multi- scale clipping method was used to augment the data, forming data sets of 2x, 4x and 8x augmentation.

Step 3 : Divide the data set into training set and test set in proportion

Step 4 : VGG convolutional neural network was used to train the model on the training set, and the neural network prediction model of depression was obtained.

Step 5 : Deploy the trained model into a small EEG detection device or wearable device for depression detection.

## 4 Results and discussions

The experimental software in this paper runs on Windows 10 64-bit operating system and is built using Python3.6 and Keras deep learning library. The hardware is Intel Core i7-10875H CPU and Nvidia RTX 2060 GPU. In the experiments. The dataset is divided into 90% training set and 10% test set. The number of training iterations is 20, and the learning rate is set to 0.0001.

The loss function uses the cross-entropy loss function, and the definition of the cross-entropy loss function is shown in Formula 7.

$$Loss=\frac{1}{n}\underset{i}{\sum }\overset{m}{\underset{c=1}{\sum }}y_{ic}\log\left(p_{ic}\right)$$ (7)

where n is the total number of samples. m is the number of categories. $y_{ic}$ is symbol function (0 or 1), the value is 1 if the true class of sample i is equal to c , otherwise is 0. $p_{ic}$ observes the predicted probability of sample i for category c .

Accuracy is used as an evaluation index to evaluate the diagnostic results. Accuracy refers to the proportion of correct results obtained by classification in the total number in a given test set. In the classification, if one category is defined as positive, other categories are negative. Accuracy is defined as Formula 8.

$$Accuracy=\frac{TP+TN}{TP+TN+FP+FN}$$ (8)

where TP is the number of positive classes predicted to be positive; FP is the number of negative categories predicted to be positive; TN is the number of negative categories predicted to be negative; FN is the number of positive classes predicted to be negative.

**FIGURE 10:**
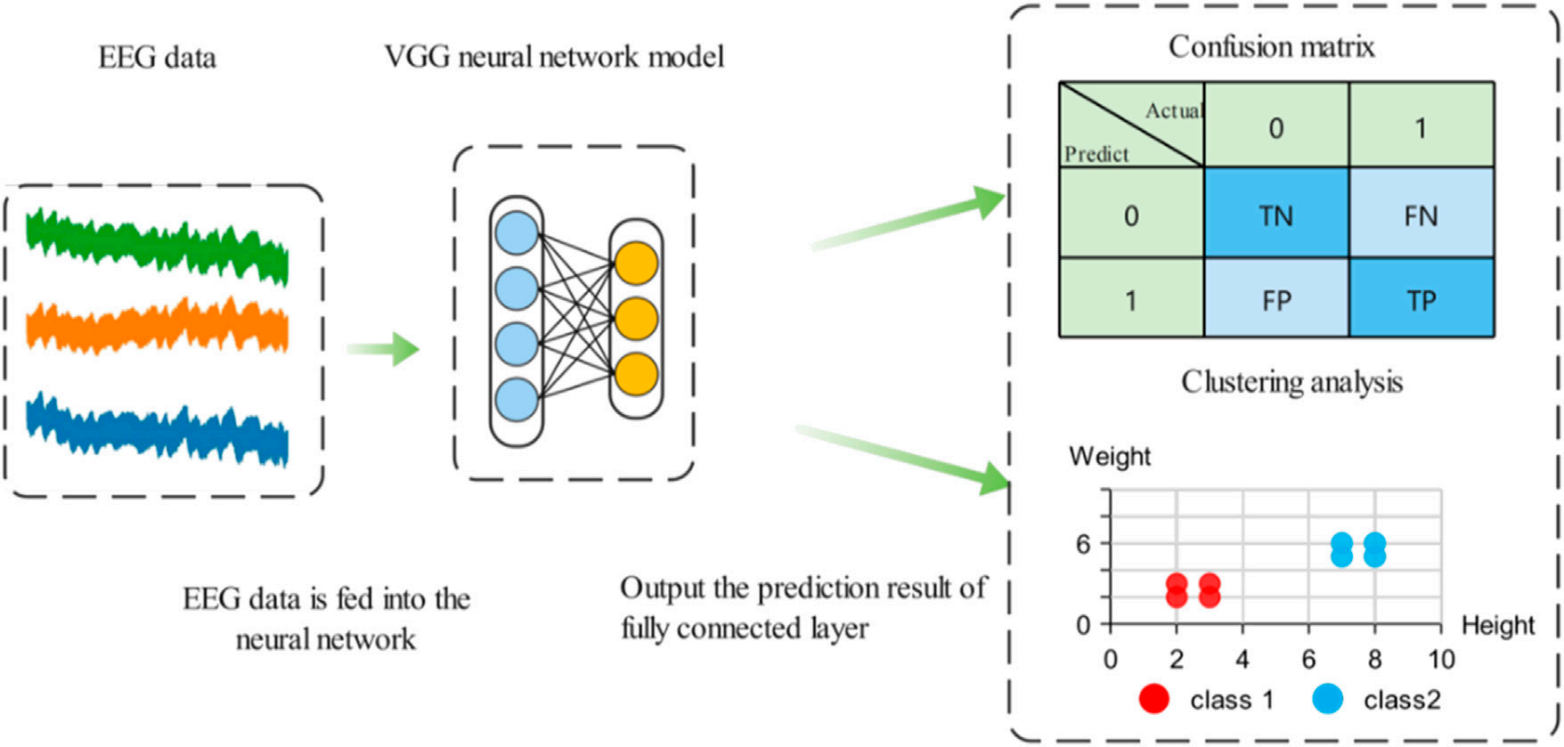
Confusion matrix and t-SNE clustering analysis.

Results visualization techniques can better observe experimental results and find classification errors between categories. Confusion matrix and t-SNE technique are used to visualize the prediction results of the model, and the specific process is shown in Figure 10 (Simonyan and Zisserman, 2014).

### 4.1 MODMA datasets

#### 4.1.1 Multi-channel data fusion

Using the proposed data processing method, the 3-channel EEG dataset was fused into a 2D image. To verify the effectiveness of multi-channel EEG signal fusion, a single- and dual-channel comparative dataset was added. The sample sizes of the three datasets are shown in Table 4, where HC stands for Healthy Control. MDD stands for Major Depression Disorder.

**TABLE 4:** 1-channel, 2-channel and 3-channel 2D MODMA datasets.

Data 1	One channel		Two channel		Three channel	
	Train set samples	Test set samples	Train set samples	Test set samples	Train set samples	Test set samples
HC	3776	419	3777	419	3772	419
MDD	3393	377	3392	376	3389	376

The curve and accuracy of the loss function obtained by training are shown in Supplementary Figure S11. Table 5 are the comparison of the final loss rate and accuracy. It can be seen from the results that with the increase of the number of fusion channels in the sample, the loss rate obtained gradually decreases and the accuracy rate increases. Compared with 65.49% of one channel, this is a big increase. By observing the confusion matrix in Supplementary Figure S12 and the clustering result in Supplementary Figure S13, it can be seen that the discrimination degree of three channels is also higher and the effect is better.

**TABLE 5:** Loss function and accuracy.

Channel	Loss function	Accuracy(%)
1-channel	0.5971	66.41
2-channel	0.3302	83.20
3-channel	0.1779	94.01

#### 4.1.2 Multi-channel data fusion and clipping augmentation

In order to verify the effect of clipped enhanced data on the performance of CNN, first of all, new datasets AU-2, AU-4 and AU-8 are obtained after augmenting data set 1,2, 4, and 8 times, the unaugmented three-channel fusion data set is called AU-N, and then these data sets are input into CNN depression diagnosis classification respectively. The data set division of the two data types is shown in Table 6.

**TABLE 6:** Multi-channel data fusion and clipping augmentation of MODMA datasets.

Data1	AU-N		AU-2		AU-4		AU-8	
	Train set samples	Test set samples	Train set samples	Test set samples	Train set samples	Test set samples	Train set samples	Test set samples
HC	3772	419	7515	835	14976	1664	29603	3289
MDD	3389	376	6757	750	13437	1493	26636	2959

The curve and accuracy of loss function obtained by training after clipping augmentation are shown in Supplementary Figure S14. Table 7 is the final comparison of loss rate and accuracy. Compared with the data set without augmentation, the accuracy reaches 99.36% after 8 times augmentation. By observing the confusion matrix in Supplementary Figure S15 and the clustering results in Supplementary Figure S16, as the multiple of data augmentation increases, the model classification after training becomes more accurate, and the feature clustering distinction between depressed patients and normal people is also greater.

**TABLE 7:** Loss function and accuracy of AU-N, AU - 2, AU - four and AU—8 data set.

AU	Loss function	Accuracy(%)
AU-N	0.1428	94.01
AU-2	0.0679	97.83
AU-4	0.0325	98.88
AU-8	0.0120	99.63

### 4.2 Depression rest data set

In order to verify the universality of the method, new data sets AU-2, AU-4 and AU-8 were obtained after 2, 4 and 8 augmentation of dataset 2. The non-augmented three-channel fusion data set was called AU-N. These data sets were generated by fusion of 3-channel EEG signals using multi-channel data fusion and clipping augmentation method and then are input into CNN respectively for diagnostic classification of depression. The data set division of the four data types is shown in Table 8.

**TABLE 8:** Multi-channel data fusion and clipping augmentation of Depression Rest data set.

Data2	AU-N		AU-2		AU-4		AU-8	
	Train set samples	Test set samples	Train set samples	Test set samples	Train set samples	Test set samples	Train set samples	Test set samples
0–13	536	59	1071	119	2138	237	4266	474
14–19	522	58	1038	115	2072	230	4133	459
20–28	529	58	1053	117	2107	234	4201	466
29–63	521	57	1014	115	2077	230	4141	460

The curve and accuracy of loss function obtained by training after clipping augmentation are shown in Supplementary Figure S17. Table 9 is the final comparison of loss rate and accuracy. Due to the small number of samples in the original data set of data set 2, after 8 times of augmentation, the accuracy of data set two reached 93.97% compared with the data set without augmentation, which was greatly improved compared with 48.66% without data cutting augmentation. By observing the confusion matrix in Supplementary Figure S18 and the clustering results in Supplementary Figure S19, as the multiple of data augmentation increases, the model classification after training is more accurate, and the feature clustering differentiation of different depression degrees is also greater.

**TABLE 9:** Loss function and accuracy of AU-N, AU-2, AU-4 and AU–8 data set.

AU	Loss function	Accuracy(%)
AU-N	1.0359	48.66
AU-2	0.8914	64.73
AU-4	0.4443	86.96
AU-8	0.2325	93.97

## 5 Conclusion

When the EEG acquisition device collects the EEG, due to different collection equipment, different patients, different collection conditions, etc., will lead to poor consistency of the collected data, which will affect the feature extraction and disease diagnosis. While, not all of the multi-channel EEG signals are depression-related, and collecting all of the channel information is not conducive to data collection, nor is it conducive to extracting depression-related features from EEG. According to these conditions, an EEG diagnosis method for depression was proposed based on multi-channel data fusion and clipping augmentation and convolutional neural network, which realized the diagnosis of depression after selecting single and multiple channels of EEG signal fusion. Firstly, in the case of data without clipping augmentation, the method is to convert multi-channel fusion into two-dimensional image. Three data sets of one, two and three channels were obtained respectively. The data sets were input into the VGG neural network for training. The training results on the two data sets showed that the more the number of channels fused in the data set, the higher the accuracy of the trained model was, and the more stable the model was. Then, the two data sets were clipping augmented, and each data set was expanded by 2, 4, and 8 times respectively. In dataset 1, the accuracy was 95.44% without fusion enhancement, and the accuracy was 97.83%, 98.88% and 99.63% after fusion enhancement by 2, 4, and 8 times, respectively. It can be seen that the accuracy has been greatly improved after data augmentation. Data set two is expanded by 2, 4, and 8 times to obtain 64.73%, 86.96% ,and 93.97%, respectively, which are greatly improved compared with without augmentation. It can be seen that when training neural network in data set with small sample size, multi-channel data fusion and clipping augmentation can alleviate the performance degradation of CNN due to small sample size and complex data. The results of the two data sets show that the combination of multi-channel data fusion and multi-scale clipping augmentation can make full use of multi-channel data in EEG, reduce the complexity of feature extraction, and quickly diagnose depression. The proposed method has low complexity and is suitable for multi-channel EEG diagnosis of depression, and has strong robustness and effectiveness.

## Data Availability

Publicly available datasets were analyzed in this study. This data can be found here: http://modma.lzu.edu.cn/data/index/

## Supplementary material

Supplementary Figure S11 Results of 1-channel dataset, 2-channel dataset and 3-channel dataset: **(A)** Train loss function, **(B)** Val loss function, **(C)** Train accuracy, **(D)** Val accuracy.

Supplementary Figure S12 Confusion matrices: **(A)** 1-channel dataset, **(B)**2-channel dataset, **(C)** 3-channel dataset.

Supplementary Figure S13 Cluster analysis: **(A)** 1-channel dataset, **(B)** 2-channel dataset, **(C)** 3-channel dataset.

Supplementary Figure S14 Results of AU-N, AU—2, AU - four and AU - 8 dataset: **(A)** Train loss function, **(B)** Val loss function, **(C)** Train accuracy, **(D)** Val accuracy.

Supplementary Figure S15 Confusion matrices: **(A)** AU-N dataset, **(B)** AU - two dataset, **(C)** AU—four dataset, **(D)** AU-8 dataset.

Supplementary Figure S16 Cluster analysis: **(A)** AU-N dataset, **(B)** AU-2 dataset, **(C)** AU-4 dataset, **(D)** AU-8 dataset.

Supplementary Figure S17 Results of AU-N, AU—2, AU—4 and AU—8 dataset: **(A)** Train loss function, **(B)** Val loss function, **(C)** Train accuracy, **(D)** Val accuracy.

Supplementary Figure S18 Confusion matrices: **(A)** AU-N dataset, **(B)** AU-2 dataset, **(C)** AU-4 dataset, **(D)** AU-8 dataset.

Supplementary Figure S19 Cluster analysis: **(A)** AU-N dataset, **(B)** AU-2 dataset, **(C)**AU-4 dataset, **(D)** AU-8 dataset.
