# Supplementary material for: EEG diagnosis of depression based on multi-channel data fusion and clipping augmentation and convolutional neural network
Source: Front Physiol. 2022 Oct 20;13:1029298. doi: 10.3389/fphys.2022.1029298 (PMC9632488; doi:10.3389/fphys.2022.1029298)
Supplement: Supplementary file 1 [file DataSheet1.pdf]

Supplementary file

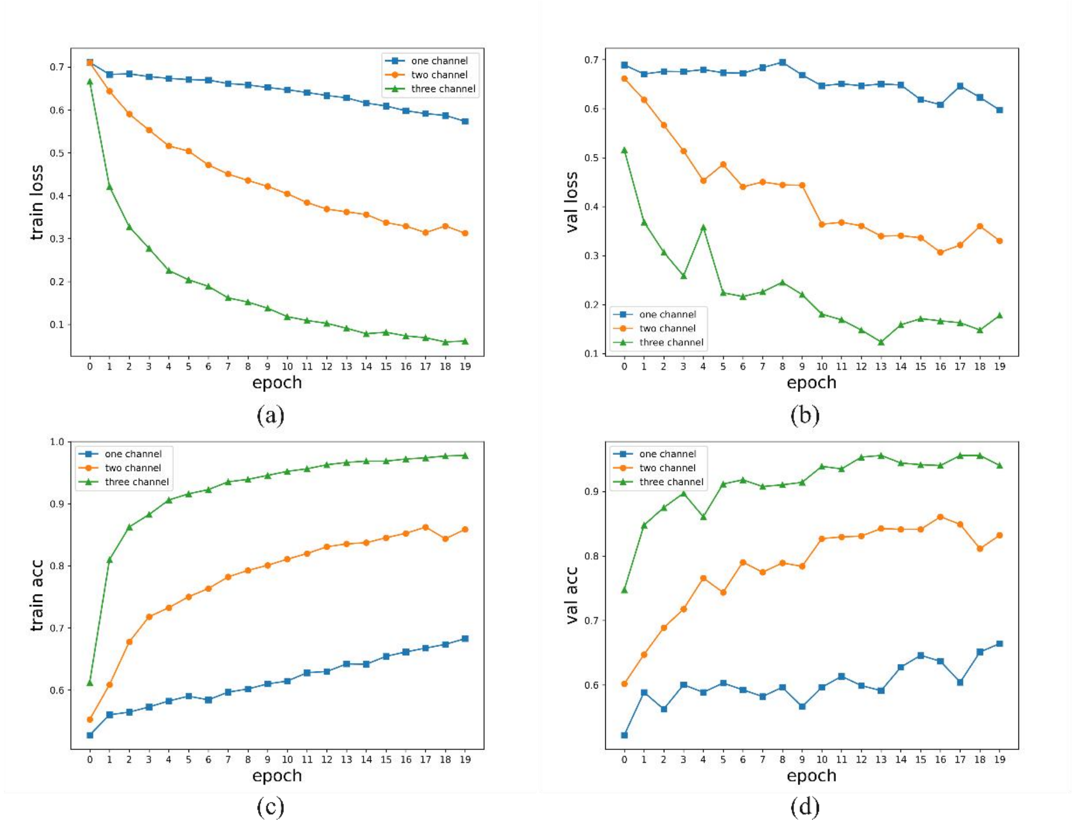

Figure 11 Results of 1-channel dataset, 2-channel dataset and 3-channel dataset: (a) Train loss function, (b) Val loss function, (c) Train accuracy, (d) Val accuracy

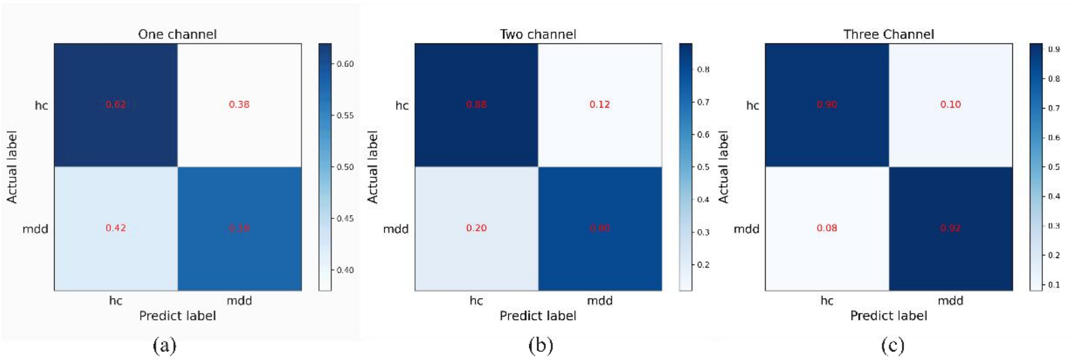

Figure 12 Confusion matrices: (a) 1-channel dataset, (b)2-channel dataset, (c) 3-channel dataset

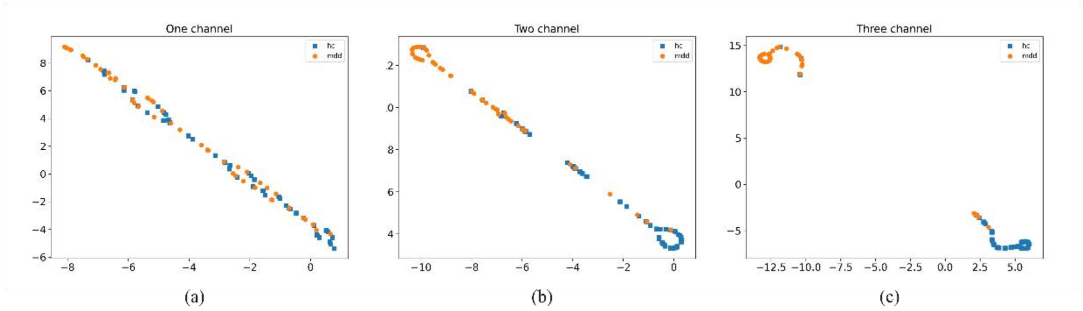

Figure 13 Cluster analysis: (a) 1-channel dataset, (b) 2-channel dataset, (c) 3-channel dataset

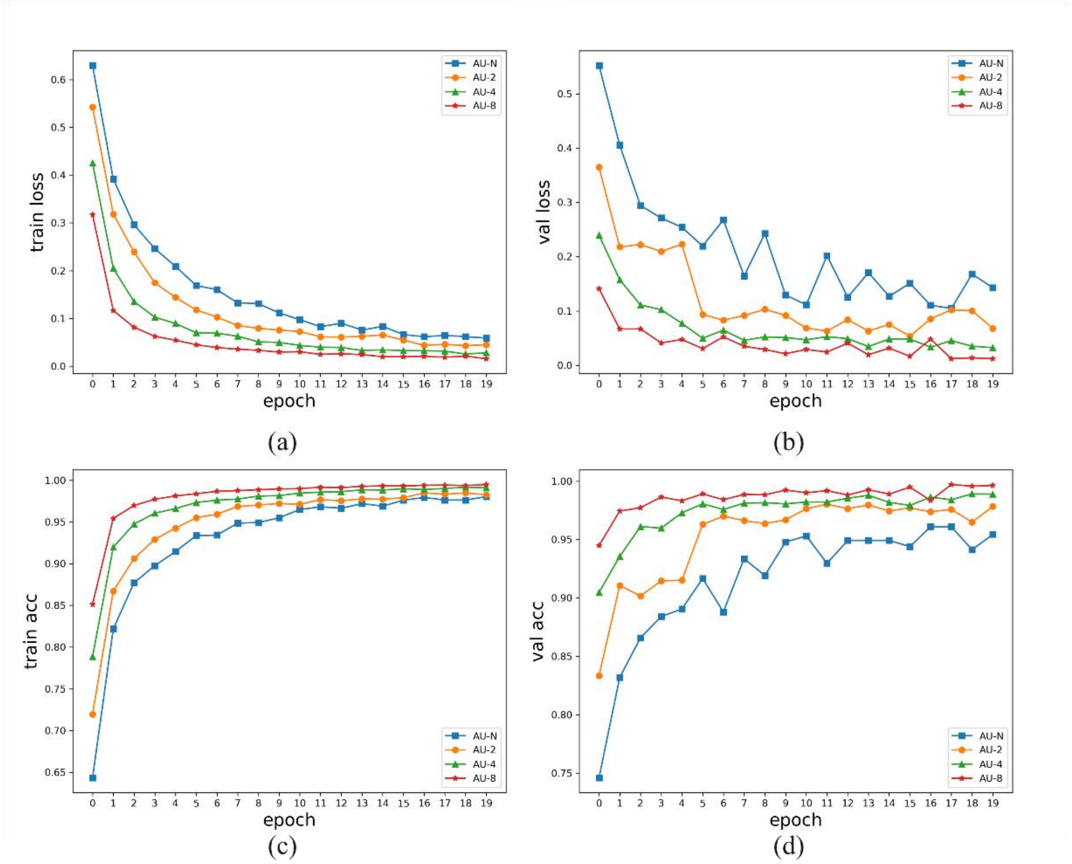

Figure 14 Results of AU-N, AU - 2, AU - 4 and AU - 8 dataset: (a) Train loss function, (b) Val loss function, (c) Train accuracy, (d) Val accuracy.

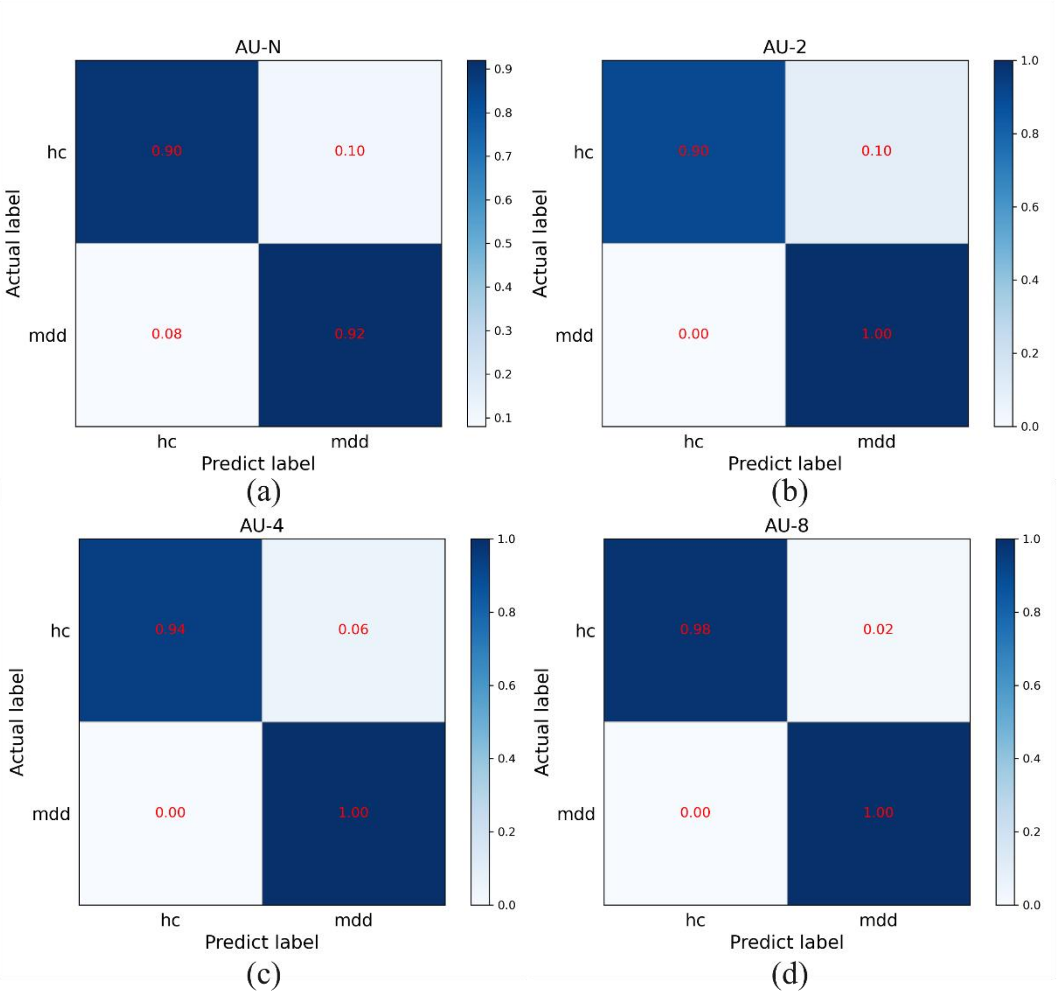

Figure 15 Confusion matrices: (a) AU-N dataset, (b) AU - 2 dataset, (c) AU - 4 dataset, (d) AU-8 dataset.

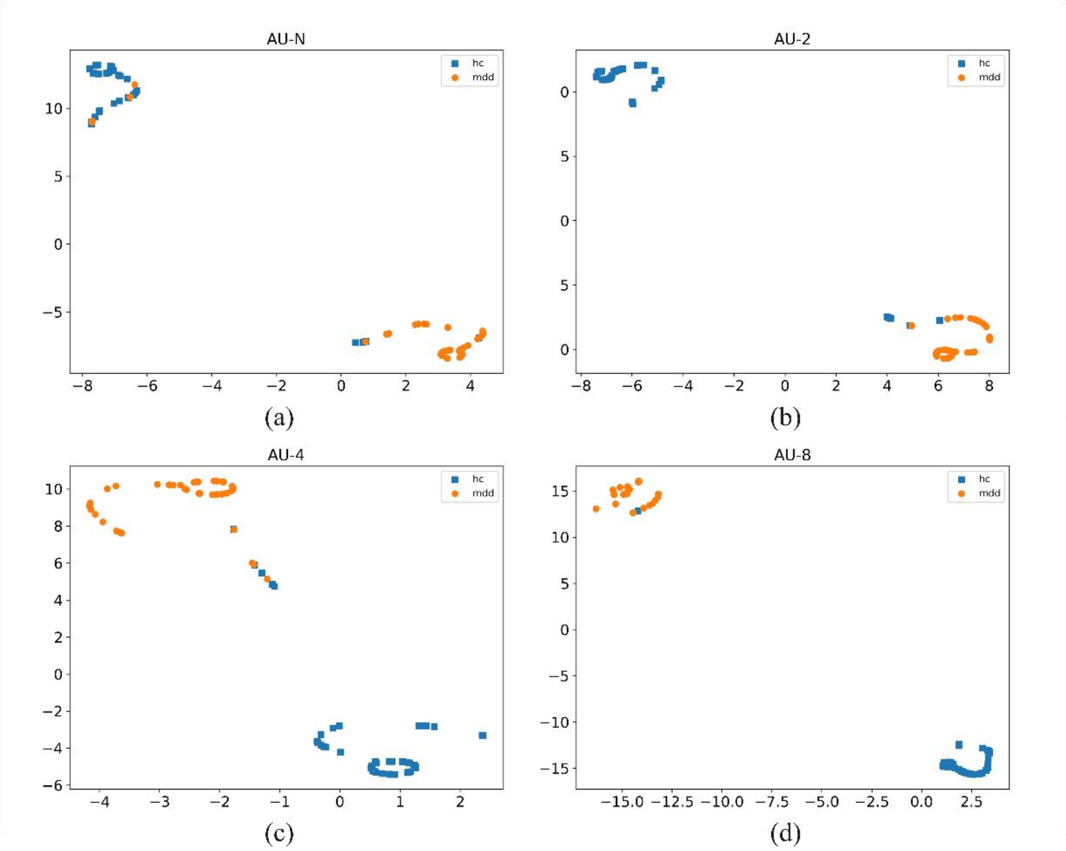

Figure 16 Cluster analysis: (a) AU-N dataset, (b) AU-2 dataset, (c) AU-4 dataset, (d) AU-8 dataset

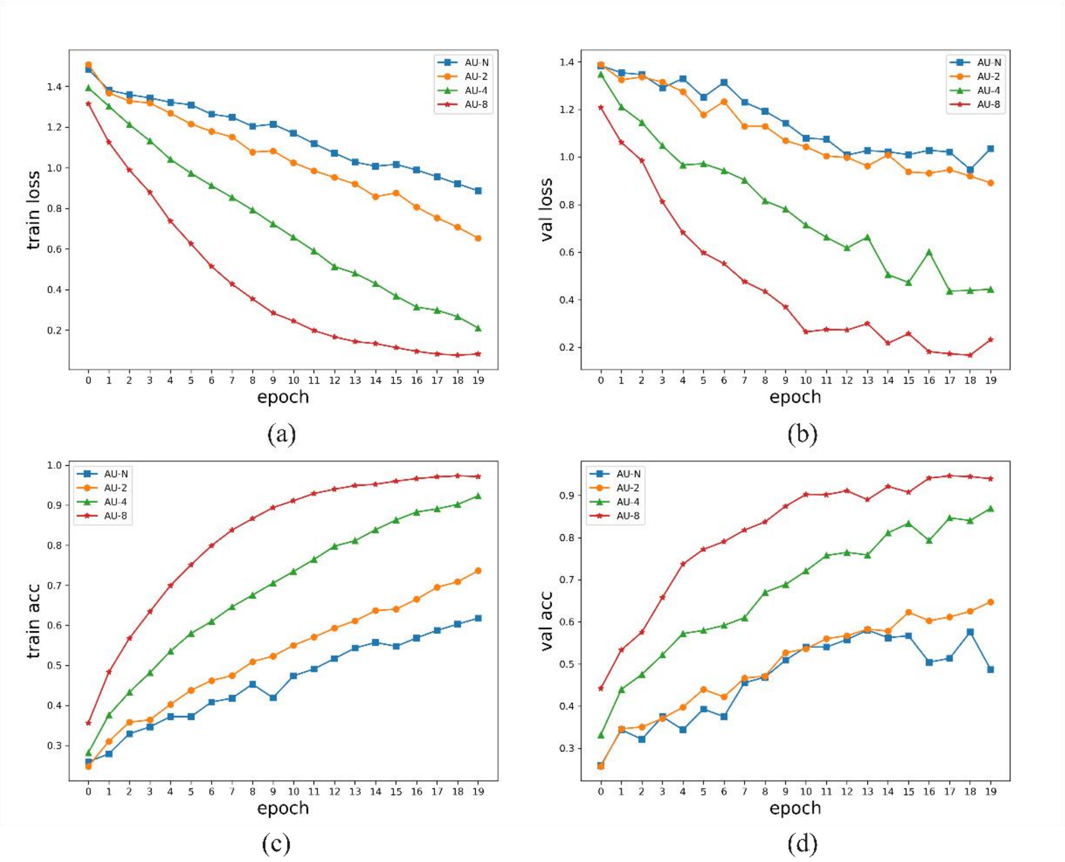

Figure 17 Results of AU-N, AU - 2, AU - 4 and AU - 8 dataset: (a) Train loss function, (b) Val loss function, (c) Train accuracy, (d) Val accuracy.

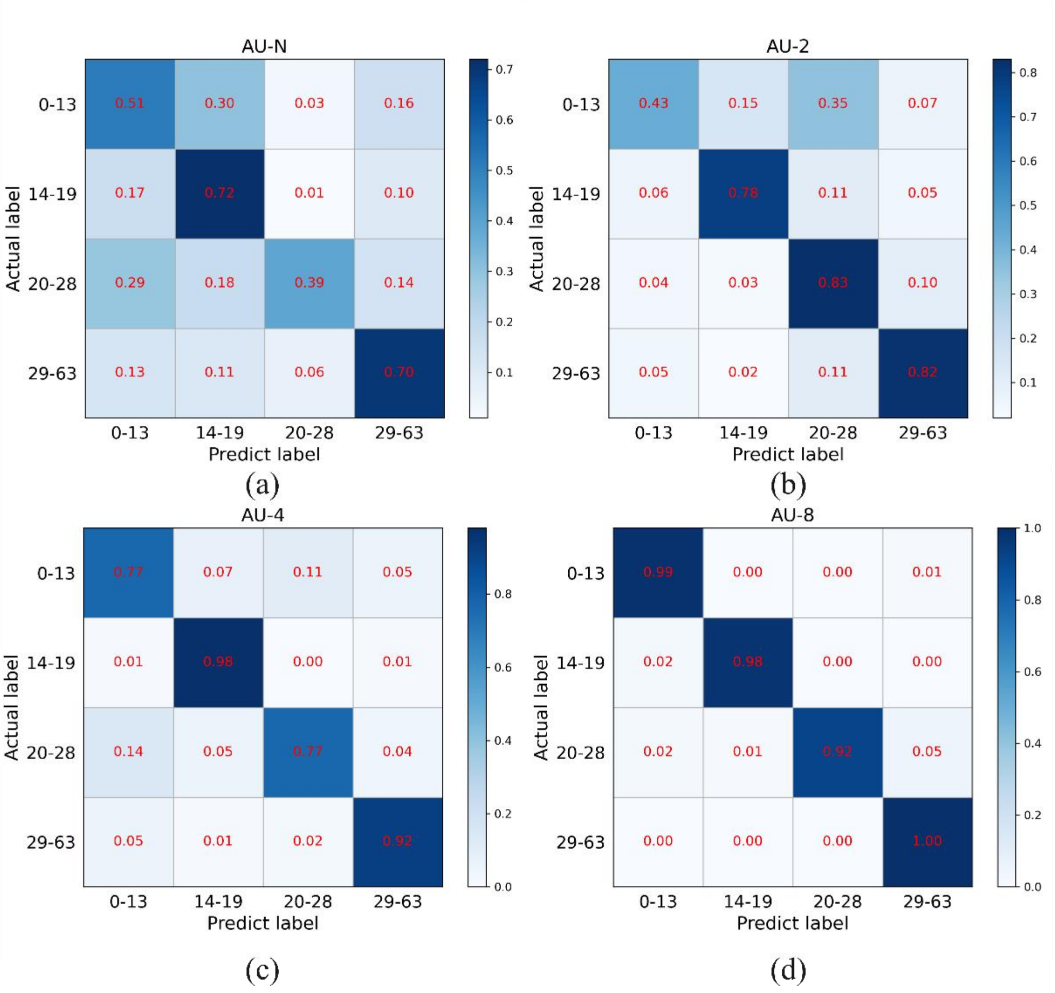

Figure 18 Confusion matrices: (a) AU-N dataset, (b) AU-2 dataset, (c) AU-4 dataset, (d) AU-8 dataset.

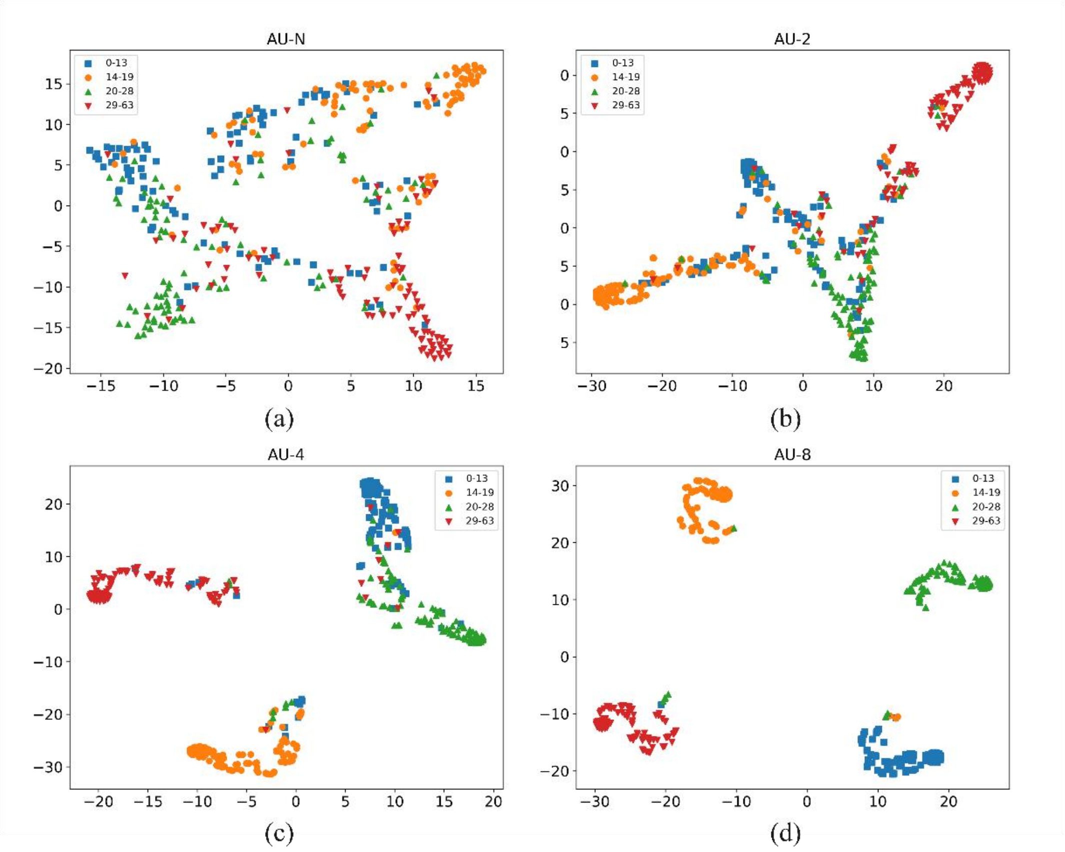

Figure 19 Cluster analysis : (a) AU-N dataset, (b) AU-2 dataset, (c)AU-4 dataset, (d) AU-8 dataset.
